# Lemuel Matthews Griffiths

**Published:** 1924-10

**Authors:** 


### IN MEMORIAM.

### Lemuel Matthews Griffiths, M.R.C.S., L.R.C.P.

The Bristol Medico-Chirurgical Society mourns the loss of Lemuel Matthews Griffiths, who died on September 22nd, in his eightieth year, an Ex-President and one of its earliest and most active members. With this issue already in the press it is quite impossible to do justice to his memory or give adequate expression to the well-earned gratitude of his medical confrères in Bristol for the many years of splendid service rendered towards founding, establishing and developing both our Medical Library and also this Journal.

The recital of even this one aspect of the life's activities of one so beloved by all who knew him demands more time than is now available. But unknown to most of his contemporaries, he also devoted much of his time and energies to sociological work in the poorer districts of our City, while he attained a world-wide reputation amongst Shakespearean scholars.

Our readers will be glad to hear that his close friend, Dr. Alexander Smith, has kindly undertaken to contribute a full obituary notice in our next issue. Nevertheless, we cannot refrain from noting the passing hence of one who has laid the whole Medical profession and more particularly the Society and its Journal under deep and lasting indebtedness.

